# Supplementary material for: Relationship between Dose Prescription Methods and Local Control Rate in Stereotactic Body Radiotherapy for Early Stage Non-Small-Cell Lung Cancer: Systematic Review and Meta-Analysis
Source: Cancers (Basel). 2022 Aug 5;14(15):3815. doi: 10.3390/cancers14153815 (PMC9367274; doi:10.3390/cancers14153815)
Supplement: Supplementary file 1 [file cancers-14-03815-s001.zip › cancers-1796835-supplementary.pdf]

Relationship between prescription methods and local control rate in stereotactic body radiotherapy for early-stage non-small cell lung cancer: systematic review and meta-analysis

Takahisa Eriguchi et al.

Supplementals

Table S1. Meta-analyses Of Observational Studies in Epidemiology checklist.

Reporting Criteria	Reported (Yes/No)	Reported on Page No.
Reporting of Background		
Problem definition	Yes	Introduction, 1st paragraph
Hypothesis statement	Yes	Introduction, 1st paragraph
Description of Study Outcome(s)	Yes	Introduction, 2nd paragraph
Type of exposure or intervention used	Yes	Introduction, 2nd paragraph
Type of study design used	Yes	Introduction, 2nd paragraph
Study population	Yes	Introduction, 2nd paragraph
Reporting of Search Strategy		
Qualifications of searchers (eg, librarians and investigators)	Yes	Materials and methods, Data extraction
Search strategy, including time period included in the synthesis and keywords	Yes	Materials and methods, Study search
Effort to include all available studies, including contact with authors	Yes	Materials and methods, Study search
Databases and registries searched	Yes	Materials and methods, Study search & Supplemental Table 3
Search software used, name and version, including special features used (eg, explosion)	Yes	Materials and methods, Study search & Supplemental Table 3
Use of hand searching (eg, reference lists of obtained articles)	Yes	Materials and methods, Study search
List of citations located and those excluded, including justification	No	Simply explained in PRISMA flow chart
Method for addressing articles published in languages other than English	Yes	Materials and methods, Study selection- design
Method of handling abstracts and unpublished studies	Yes	Materials and methods, Study selection- design
Description of any contact with authors	Yes	Materials and methods, Study search
Reporting of Methods		
Description of relevance or appropriateness of studies assembled for assessing the hypothesis to be tested	Yes	Materials and methods, Data extraction
Rationale for the selection and coding of data (eg, sound clinical principles or convenience)	Yes	Materials and methods, Data extraction
Documentation of how data were classified and coded (eg, multiple raters, blinding, and interrater reliability)	Yes	Materials and methods, Data extraction
Assessment of confounding (eg, comparagraphbility of cases and controls in studies where appropriate	Yes	Materials and methods, Data extraction
Assessment of study quality, including blinding of quality assessors; stratification or regression on possible predictors of study results	Yes	Materials and methods, Study quality assessment
Assessment of heterogeneity	Yes	Materials and methods, Study quality assessment
Description of statistical methods (eg, complete description of fixed or random effects models, justification of whether the chosen models account for predictors of study results, dose-response models, or cumulative meta-analysis) in sufficient detail to be replicated	Yes	Materials and methods, statistics
Provision of appropriate tables and graphics	Yes	Results

Reporting of Results		
Table giving descriptive information for each study included	Yes	Table 1
Results of sensitivity testing (eg, subgroup analysis)	Yes	Figure 3, Table2
Indication of statistical uncertainty of findings	Yes	Figure 3, Table2
Reporting of Discussion		
Consideration of alternative explanations for observed results	Yes	Discussion, 2-6th paragraph
Generalization of the conclusions (ie, appropriate for the data presented and within the domain of the literature review)	Yes	Conclusions
Guidelines for future research	Yes	Discussion, 7th paragraph
Disclosure of funding source	Yes	Footnote

Table S2. Inclusion and exclusion criteria.

Inclusion criteria
SBRT for peripheral NSCLC
LC and dose prescription provided
**Exclusion criteria**
Central lesion*
Metastatic lung tumor
Small cell lung cancer
Studies limited to
Stage III, IV
T3, T4*
postoperative recurrence
specific pathology
ground glass opacity
Concurrent chemotherapy
Charged particle radiotherapy
Fraction number > 10
Median f/u < 24 months
Patient number < 25
Review or case report
Non-English study

Abbreviations: SBRT = stereotactic body radiotherapy; NSCLC = non-small cell lung cancer; LC = local control; f/u = follow-up. * Including less than 10% of central lesion or T3, T4 tumors are permitted.

Table S3. Search formulas.

Database and search formula	N
PubMed	1350
#1 (NSCLC OR non-small cell lung cancer OR ((lung OR pulmonary) AND (cancer OR carcinoma OR malignancy OR tumor OR neoplasm OR neoplasms OR carcinomas OR malignancies)))<br>#2 (SBRT[title] OR SRT[title] OR SABR[title] OR stereotactic[title])<br>#3 (local control OR local recurrence OR local relapse OR local progression)<br><br>#4 #1 and #2 and #3	

Table S4. Newcastle-Ottawa Scale score.

1st author	Total	Representative (0 or 1)	Selection on non-exposed (0 or 1)	Ascertainment (0 or 1)	Demonstration (0 or 1)	Comparability (0 or 2)	Assessment (0 or 1)	Long enough (0 or 1)	Adequate followup (0 or 1)
Nagata	5	1	0	1	0	0	1	1	1
Salazar	5	1	0	1	0	0	1	1	1
Takeda	5	1	0	1	0	0	1	1	1
Fakiris	5	1	0	1	0	0	1	1	1
Fakiris	5	1	0	1	0	0	1	1	1
Ricardi	5	1	0	1	0	0	1	1	1
Brown	5	1	0	1	0	0	1	1	1
Grills	5	1	0	1	0	0	1	1	1
Timmerman	6	1	0	1	1	0	1	1	1
Shirata	5	1	0	1	0	0	1	1	1
Shirata	5	1	0	1	0	0	1	1	1
Inoue	5	1	0	1	0	0	1	1	1
Suzuki	5	1	0	1	0	0	1	1	1
Hamaji	5	1	0	1	0	0	1	1	1
Lindberg	5	1	0	1	0	0	1	1	1
Shibamoto	5	1	0	1	0	0	1	1	1
Hayashi	5	1	0	1	0	0	1	1	1
Nagata	6	1	0	1	1	0	1	1	1
Shaverdian	5	1	0	1	0	0	1	1	1
Tsurugai	5	1	0	1	0	0	1	1	1
Navarro-Martin	5	1	0	1	0	0	1	1	1
Mancini	5	1	0	1	0	0	1	1	1
Aoki	5	1	0	1	0	0	1	1	1
Sun	6	1	0	1	1	0	1	1	1
Miyakawa	6	1	0	1	1	0	1	1	1
Lee	5	1	0	1	0	0	1	1	1
Raghavan	5	1	0	1	0	0	1	1	1
Cummings	5	1	0	1	0	0	1	1	1
Cummings	5	1	0	1	0	0	1	1	1
Karasawa	5	1	0	1	0	0	1	1	1

Menoux	5	1	0	1	0	0	1	1	1
Wegner	5	1	0	1	0	0	1	1	1
Tsurugai	5	1	0	1	0	0	1	1	1
Tsurugai	5	1	0	1	0	0	1	1	1
Ball	6	1	0	1	1	0	1	1	1
Weiss	5	1	0	1	0	0	1	1	1
Shu	5	1	0	1	0	0	1	1	1
Duvergé	5	1	0	1	0	0	1	1	1

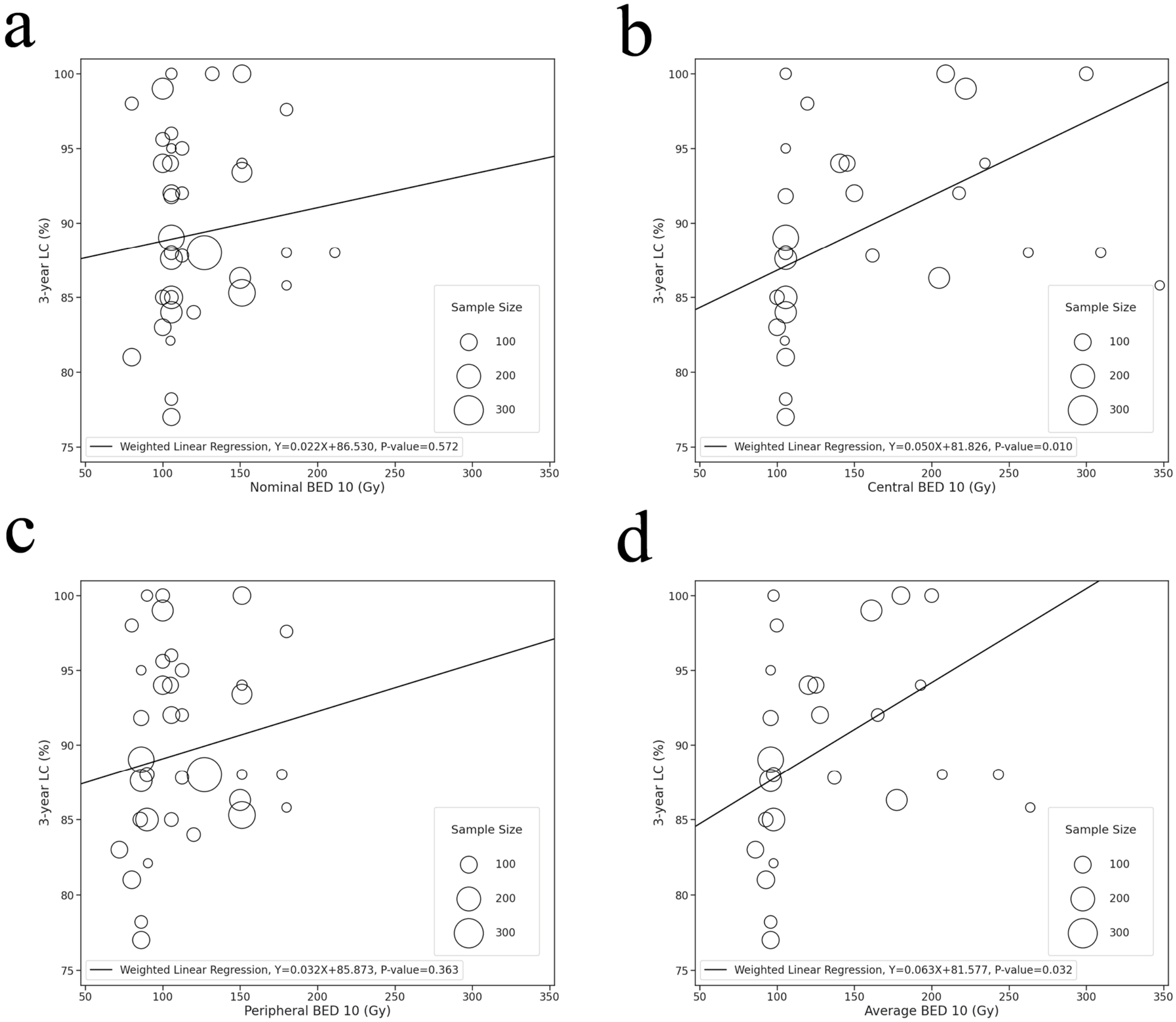

Figure S1. The 3-year local control rate according to (a) nominal BED, (b) central BED, (c) peripheral BED, and (d) average BED with weighted Spearman correlation.
